# Patterning of Particles and Live Cells at Single Cell Resolution

**DOI:** 10.3390/mi11050505

**Published:** 2020-05-15

**Authors:** Adar Hacohen, Hadass R. Jessel, Alon Richter-Levin, Orit Shefi

**Affiliations:** 1 The Mina & Everard Goodman Faculty of Life Sciences, Bar-Ilan University, Ramat Gan 5290002, Israel; hadassrjessel@gmail.com; 2 The Faculty of Engineering, Bar Ilan University, Ramat Gan 5290002, Israel; alonrichter@gmail.com (A.R.-L.); oshefi@gmail.com (O.S.); 3 Bar Ilan Institute of Nanotechnologies and Advanced Materials, Bar Ilan University, Ramat Gan 5290002, Israel

**Keywords:** micropatterning, micromanipulator, single cell resolution, complementary DNA sequences

## Abstract

The ability to manipulate and selectively position cells into patterns or distinct microenvironments is an important component of many single cell experimental methods and biological engineering applications. Although a variety of particles and cell patterning methods have been demonstrated, most of them deal with the patterning of cell populations, and are either not suitable or difficult to implement for the patterning of single cells. Here, we describe a bottom-up strategy for the micropatterning of cells and cell-sized particles. We have configured a micromanipulator system, in which a pneumatic microinjector is coupled to a holding pipette capable of physically isolating single particles and cells from different types, and positioning them with high accuracy in a predefined position, with a resolution smaller than 10 µm. Complementary DNA sequences were used to stabilize and hold the patterns together. The system is accurate, flexible, and easy-to-use, and can be automated for larger-scale tasks. Importantly, it maintains the viability of live cells. We provide quantitative measurements of the process and offer a file format for such assemblies.

## 1. Introduction

The manipulation and patterning of individual micro-objects is a critical technology for micro-assembly [1,2] and many biomedical applications [3,4]. Biological elements, cells, tissues, and organs are organized in specific microscopic structures, with many kinds of cells positioned and arranged at multi-scale resolutions. Each plays a role at its respective position. Therefore, an important goal in tissue engineering and the study of the replication of tissue micro-environments is the development of a technique to position different types of cells and extracellular elements precisely into a specific predefined position with a corresponding resolution, keeping the cells viable and functioning [5,6]. In addition, such a method will make it possible to study cell biology at a single cell level, by examining single cell interactions with drugs, growth factors, or external stimuli. Studying cell migration, cell signaling, and drug screening also requires spatial positioning that includes the ability of capturing, transporting, and locating single cells in desired orientations [7,8,9]. An important challenge of cellular organization is the formation of neural networks [10]. For example, to control and direct neuronal growth during regeneration, 2D and 3D scaffolds are designed. Fabrication methods range from photolithography [11,12,13] to self-assembly [14]. Generally, there are two approaches to control cell positioning and growth. The first, is a top-down approach, the use of cellular interfaces with physical and chemical characteristics [15]. The second approach is a bottom-up approach, the micro-positioning of discrete objects that are built into a complex structure or organization. The principle of the bottom-up approach is to pattern individual components of a tissue according to a predefined pattern, which will guide the maturation of cell self-sorting and self-assembly capabilities, as well as the morphogenesis mechanism [16].

A variety of particles and cell patterning methods have been demonstrated [15,17], such as dielectrophoresis [18], magnetic force-based methods [19,20], acoustic traps [21], and hydrodynamic flows [22]. These techniques provide high throughput, but lack the flexibility or the spatial resolution necessary for controlling individual cells. There were a few attempts to adapt some of these methods to single cell resolution by using a low initial sample concentration, or by decreasing the force used [23,24]. Optical tweezers [25,26,27] offer high resolution for trapping single particles, and therefore can be used for single cell patterning. However, they have a limited manipulation area, owing to tight focusing requirements.

Other methods for cell patterning include soft lithographic techniques, such as microcontact printing [28,29], where cells adhere to selectively biochemically treated areas and form the printed pattern, and microwell trapping [30], where single-cell entrapment occurs in micropores or a defined diameter after the deposition of a cell suspension before the excess medium is removed. Although single cells are trapped, these methods do not have the ability to typically position multiple cell types in precise arrangements, and offer little flexibility in cell size variation, pattern shape, or spacing.

In the bioprinting strategies, inkjet printers provide a potential strategy to control spatial positioning of cells. Different attempts to use an inkjet printer to generate droplets that contain a single cell were reported [31]. However, inkjet printers cannot position a single cell in a defined position in a resolution smaller than 10 µm, due to inaccuracy derived from the variation of the flight angle, the initial position of the cell in the droplet, and cell movement on the surface at the moment of impact. In addition, this method suffers from low cell viability impacted by shear stress, and it is not able to create dense cell patterns. Laser-assisted printing methods, e.g., laser-induced forward transfer (LIFT) and other laser-based direct writing (LDW) techniques, which use a laser beam to transfer or propel cells from one source film (donor, ribbon or target) to a receiving or acceptor substrate, enable the transfer of single cells on the substrate in a high accuracy [32,33].

Micromanipulators are devices intended to translate macroscopic movements of the human hand into microscopic movements of a fine tool held in its grasp. Therefore, they are very useful in experiments that are conducted under a microscope, in which there is a need to hold or move very small instruments or tools. Micromanipulators are used widely in electrophysiology and life sciences research, e.g., patch clamp [34], the intracytoplasmic sperm injection (ICSI) procedure [35], the micropipette aspiration of cells [36,37] and microinjection [38]. In each procedure, different tools and micropipettes are attached to the micromanipulator. For example, micromanipulators, along with microinjection systems, are typically used in combination with an injector pipette to deliver sub-milliliter volumes of fluid media into a substrate, and in combination with a holding pipette, in order to immobilize the substrate, via the application of pressure. The pressure may be generated pneumatically or hydraulically. In the ICSI procedure, one micromanipulator is equipped with a holding pipette and holds the oocyte, while a second micromanipulator is equipped with an injection pipette to inject the sperm into the oocyte. In other experiments, drugs, signaling molecules, and viral vectors have been injected into cells and tissues for the study of the mechanisms of development and disease progression [39,40,41]. More recently, and more relevantly for tissue engineering and cell patterning, micromanipulation and microinjection techniques have also been reported to be used to deliver cells [42,43] and synthetic polymer microspheres [44,45] by injection into adult tissues. However, the system was not utilized to selectively pick objects and precisely position them in a defined pattern.

In this study, we have configured a micromanipulator system equipped with a pneumatic micro-injector, coupled to a holding pipette, that has the ability to create patterns of particles and cells from multiple types with high accuracy and precision. Our method offers an advantage over other patterning techniques, as it combines single cell patterning and precise positioning with the ability to selectively choose the particles that are positioned, while keeping the cells viable. Moreover, we propose an approach to keep intact the positioned cells by oligonucleotides sequences, overcoming the need for stability. In this paper, we show a construction of two-dimensional patterns of beads and cells, opening the possibility for the complex and realistic design of tissues.

## 2. Materials and Methods

### 2.1. Micro Manipulation System

A micromanipulation station setup was constructed, including two fully motorized micromanipulators (Patch star, Scientifica, Uckfield, UK), which provide 20 mm of movement in each axis, with a 20 nm resolution throughout its range of travel. The micromanipulator was coupled with a pneumatic injector (IM–11-2, Narishige, Tokyo, Japan), equipped with coarse-fine coaxial control knobs: one for coarse pressure changes and one for fine pressure changes. In order to control the knob movement through joysticks and the software, a control cube was integrated to the system by a rack that connects the microinjecters fine knob to the control cube (Z-focus for motorised objective focusing, 1U rack and control cube). Adjustment of the microinjector to the desired response speed is done by turning the control knob on the injector to the working position, while holding down the pressure relief valve that disables pressure change. The working position of both coarse and fine knobs can be adjusted individually. To achieve faster response the working position of the control knob (course/fine) should be set towards the front, and to achieve a slower response the working position should be set towards the rear. The default working position was set to the middle between the front and the rear to obtain, on the one hand, enough range of movement and, on the other hand, a speed that can be monitored by eye. In addition, the setup includes an inverse phase contrast microscope with a magnification of 10× and 4× (Olympus, Tokyo, Japan), a camera (acA1600-60gm GigE camera, Basler, Ahrensburg, Germany), user interface (Linlab2,Scientifica, Uckfield, UK), and a Hewlett-Packard PC for automatic control. The micromanipulators are mounted on the microscopes stage. The full set up sites on a screw down table stage.

### 2.2. Selection of Capillary

For particles and cells with 30µm diameter, a Small holding micropipette, SHM, (MPH-SM-35, Origio, Målov, Denmark) was used. SHM has an angle of 35°, an inner diameter of 15–20 µm, and an outer diameter of 65–95 µm.

For particles with a diameter between less than 20 µm (and above 10 µm), a custom-made Baby holding micropipette, BHM (C130426, Origio, Målov, Denmark) was used. BHM has an angle of 30°, an inner diameter of 5–7 µm, and an outer diameter of 35 µm.

### 2.3. Cell-Holding Device

The device was designed in DS Solid Works and printed on a Stratasys Connex3 3D printer (Stratasys, Rehovot, Israel), using biocompatible MED610 material. Support resin was removed from the finished models by water jet followed by soaking in 5% NaOH for 2 hours at room temperature, and finally a thorough wash with water. 

### 2.4. Beads

Polystyrene beads (M30352, Rapp-polymere, Tubingen, Germany) and Streptavidin Sepharose beads (GE17-5113-01, Sigma-Aldrich, St. Louis, MO, USA) were suspended in Phosphate-buffered saline (PBS in concentration of 2 × 10^5^ cells/mL.

### 2.5. DNA-Coated Beads

Streptavidin Sepharose beads were attached to DNA oligonuclutides via biotin-strep covalent interaction. The two complement sequences that were used are: **A:** /5BiotinTEG/TT TTT TTT TTT TTT TTT TTT TTT TTT TTT TTT TTT TTT TTT TTT TTT TTT TTT TTT TTT TTT TTT TTT TTT TTT TTT TTT ACT GAC TGA CTG ACT GAC TG, **A’:** /5BiotinTEG/TT TTT TTT TTT TTT TTT TTT TTT TTT TTT TTT TTT TTT TTT TTT TTT TTT TTT TTT TTT TTT TTT TTT TTT TTT TTT TTT CAG TCA GTC AGT CAG TCA GT. The DNA sequences were attached to the beads by incubation of 2 h in PBS solution with the streptavidin Sepharose beads, in a ratio of 10× DNA:Streptavidin. Oligonucleotides were commercially synthesized (IDT, Coralville, IA, USA).

### 2.6. Agarose Droplets Creation

Hydrogel droplets were created by a microfluidic device (Encapsulator 1 Dolomite microfluidics). The layout of the microfluidic device includes a sample reservoir chip, a droplet chip, and a 4-way linear connector that connects the tubing to the sample reservoir chip and 3 pressure pumps, a high magnification microscope continuously monitors the device junction, a temperature control unit (TCU), and a Flow Control Center, which controls and displays the pressure/flow of each of the 3 pumps, controls the temperature of the TCU and displays camera. The microfluidic device utilizes a flow focusing junction geometry, where the droplet phase is non-wetting, while the carrier phase wets the channel surfaces. Sample preparation: an agarose solution was prepared by dissolving ultra-low gelling temperature agarose (9012-36-6, Sigma-Aldrich, St. Louis, MO, USA) in water, followed by melting above 65 °C. Final agarose concentration was 3%. A total of 100 µL of this solution was loaded to each of the sample loading ports on the reservoir chip. Temperature control was fixed to 37 °C. Pico-Surf1TM was used as the oil phase. The driving fluid used in pump s1 and pump s2 (drive the aqueous phase) was novex 7500. Flow values of the oil pump: 15 µL/min and of the 2 aqueous pumps were set to 1 µL/min. All droplets were collected and observed with a microscope. Agarose droplets after collection were uniform in their diameter. The diameter of the droplets can be controlled by the pressure parameters of the pump, and can change between 20–30 μm. Then, droplets were solidificated by cooling in 4 °C for 2 h. Oil was removed from droplets by Picobreak Dolomite protocol.

### 2.7. Cell Culture

Primary Leech Neurons culture—Ganglia were isolated from the central nervous system of adult medicinal leeches *Hirudo medicinalis.* Leeches were anesthetized on ice for 30 min before dissection, and pinned dorsal side up to a layer of sylgard on the bottom of the dissection chamber. Dissection was carried out according to an established procedure. Briefly, a longitudinal cut was made through skin and muscle layers along the dorsal midline of the leech, to expose the nerve cord and segmental ganglia. Ganglia were then dissected and moved to a Sylgard-184 petri dish containing 4 mL of room temperature-enriched Leibovitz medium (L-15 medium supplemented with 6mg/mL glucose, 0.1mg/mL gentamycin, 2mM/mL glutamine, and 2% fetal bovine serum). In total, 30 ganglia were used for this study. Next, ganglia were pinned on the sylgard layer, ventral aspect up, and the L15-enriched medium was replaced with fresh medium. Ganglia were held in the L-15-enriched medium. Ganglia were treated enzymatically with 2 mg/mL collagenase/dispase enzyme solution (Roche, Mannheim, Germany) for 1h at room temperature. Next, ganglia capsules were opened to expose the cells. Neuronal cells were rinsed and plated on 35 mm petri dish precoated with Concanavalin-A (Con-A) to facilitate cell adhesion (Sigma-Aldrich, 0.5 mg/mL). Enriched medium was added to the cultures (Leibovitz L-15 supplemented with glucose, gentamycin, glutamine, and fetal bovine serum).

Hep G2 (HB-8065, ATCC, Manassas, VA, USA) were grown in culture media Eagle’s Minimum Essential Medium (EMEM), supplemented with 10% fetal bovine serum (Gibco FBS, Thermo Fisher Scientific, Waltham, MA, USA), 1% streptomycin (Gibco), and incubated in a humidified condition with 5% CO_2_ at 37 °C. Cells at 80% confluence were trypsinized and subcultured to produce a cell suspension at an appropriate cell concentration.

HeLa cells (ATCC CCL-2) were grown in culture media (EMEM), supplemented with 10% fetal bovine serum (Gibco), 1% streptomycin (Gibco), and incubated in a humidified condition with 5% CO_2_ at 37 °C. Cells at 80%–90% confluence were trypsinized and subcultured, to produce a cell suspension at an appropriate cell concentration.

### 2.8. Cell Viability Assay

Cells were harvested and suspended on two separate positions on a dish culture with two identical concentrations (2.5 × 10^5^ cells/mL). One dish was a control, not manipulated, and the other one was manipulated. In the manipulated dish we have caught and released all cells (~100). Next, we stained the cells with Propidium iodide (PI) and Hoechst 33342 (Thermo Fisher Scientific, Waltham, MA, USA). An inverted microscope system was used (Eclipse Ti2, Nikon) to capture the PI intensity of all cells and determine viability.

### 2.9. Error Measurement

Video data was analyzed in MATLAB using a custom-written code. Particle centroids were detected once when caught in pipette and secondly after release. The Euclidean distance between the centroids was measured in pixels, and then converted into µm units.

## 3. Results

**Figure 1:**
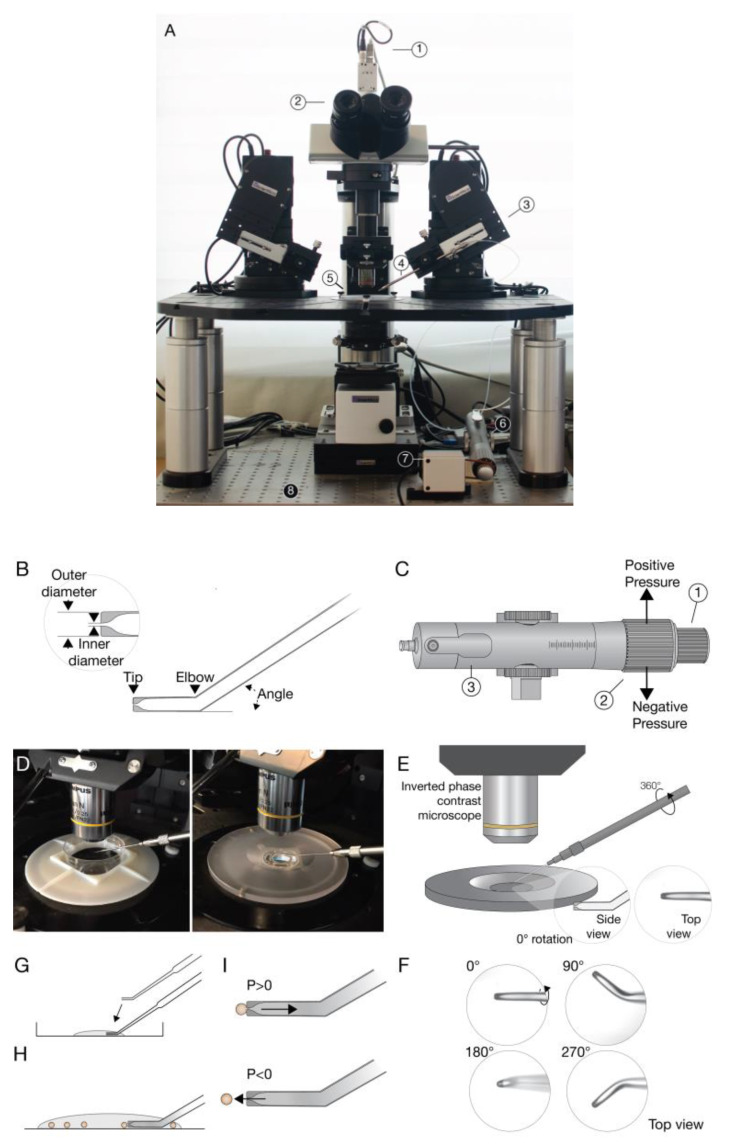
System construction and operation. (**A**) 1. Camera; 2. inverse phase contrast microscope; 3. micromanipulator; 4. microinjection holder; 5. microscope stage; 6. microinjector; 7. microinjector engine; 8. vibration isolated platform. (**B**) Holding micropipette with a flat, polished opening. Two sizes were used: a small holding micropipette (SHM) and a baby holding micropipette (BHM). The SHM has an outer diameter of 65–95 µm, and an inner diameter of 15–20 µm, and an angle of 35°. SHM is suitable for patterning of particles of 30 µm diameter. The BHM has an outer diameter of 35 µm and an inner diameter of 5–7 µm, and an angle of 30°. BHM is suitable for patterning of particles smaller than 20 µm diameter. (**C**) Microinjector:1. Pressure relief valve; 2. coarse knob; 3. fine knob (**D**) Working plate: 25 mm petri-dish placed on working plate (left). Custom CAD design of cell-holding device embedded on working plate for separation between different types of particles. Has a central well for assembly and four external wells, signed with letters for particle reservoirs (right). (**E**) Pipette approach to particle. The microinjector holder can be rotated in 360° to give different angle approaches of the micropipette. The pipette approach is monitored by the microscope that gives a top view of the pipette. The default approach (0°) is when the bent part of the pipette is parallel to the surface (**E**). A rotation of 90° (/−90°) is used for patterning, enables correct manipulation and monitoring of the process (**F**). (**G**) A drop of the particle solution is loaded onto the petri-dish, and the micropipette is moved into the drop allowing pipette to soak solution up by capillary action. (**H**) The micropipette is positioned in close proximity to a specific particle. (**I**) Positive pressure induces suction and causes the particle to be caught on the pipette, while negative pressure induces injection and leads to the release of the particle from the pipette.

A micromanipulation station setup was constructed (Figure 1A) including two fully motorized micromanipulators, coupled with a pneumatic microinjector. Holding micropipettes were connected to the microinjector and used to grab and dispense single cells (Figure 1B,C). They have a flat, polished opening, with a large ratio of outer diameter to inner diameter for maximum control, without causing any harm to the cells. In addition, the pipettes that were used have an angle in their edge to ease the access to the manipulated particles (Figure 1B). Pipette size were matched to particle size to give optimal performance, i.e., to disable the suction of the particle into the pipette and enable a precise catch and release of particles. The micromanipulators are mounted on a stage of an inverse phase contrast microscope. The stage enables flexibility in the location of the micromanipulators and integration of other devices to the system. The whole setup sits on a screw down table stage to minimize motion. All system components can be controlled manually via joysticks and automatically via the software. A working plate device for insertion of a 35 mm petri dish, on which the manipulation is performed was designed and printed (Figure 1D).

The angle approach of the pipette to the particles is an issue to be addressed. The default approach (0°) is defined as when the bent part of the pipette is parallel to the surface (Figure 1E). A rotation of 90°(/−90°) of the microinjector holder was preformed, for an optimal pipette approach that enables correct manipulation and monitoring of the process, i.e., it gives a good display of the pipette’s tip and of the particle caught and released from it (Figure 1F).

The operation of the system starts with the loading of a drop of particles in medium on the petri dish. The micropipette is moved into the drop, and allows pipette to soak the particle medium up by capillary action (Figure 1G). The movement of the pipette continues to the bottom of the dish, where the particles are placed, and is positioned in close proximity to a specific particle (Figure 1H). Then, a positive pressure is set, inducing suction of the particle to the pipette. Releasing the particle will be induced by reducing the pressure to a negative pressure (Figure 1I).

**Figure 2:**
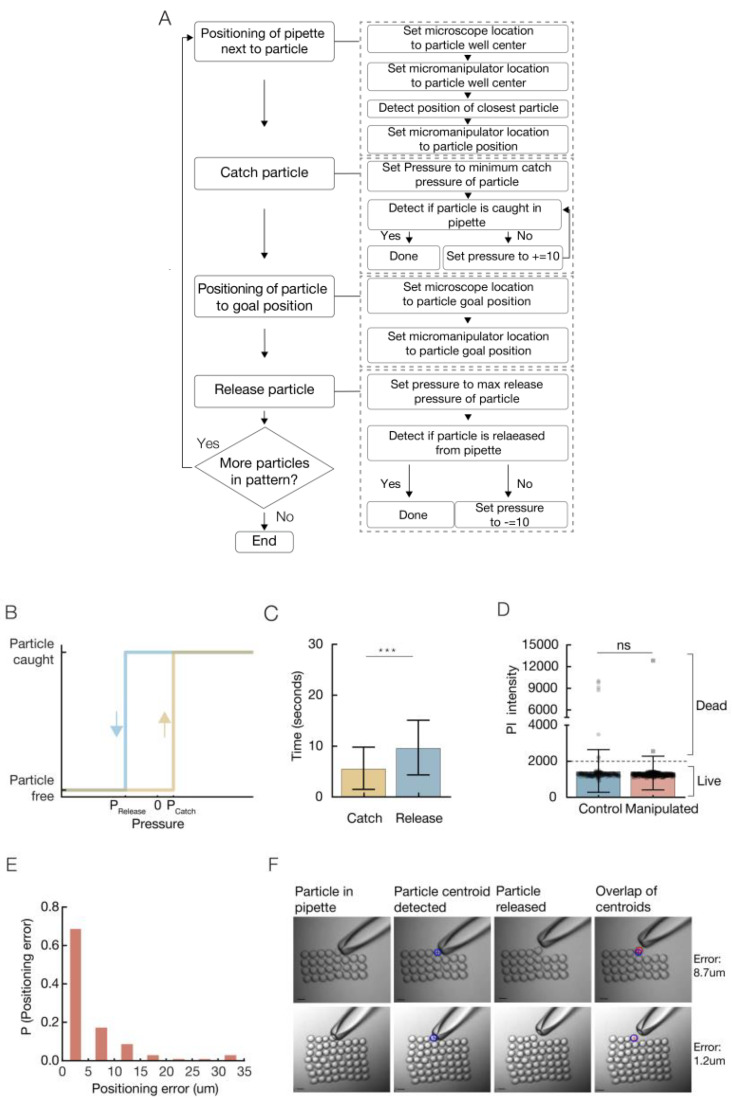
Calibration and Characterization of single particle manipulation. (**A**) Schematic overview of all steps in the assembly process. Each of the steps can be automated. (**B**) Pressure values are calibrated for each particle type. P_Catch_ is the minimum pressure that is required for the particle to be caught on the pipette when the pipette is positioned in near proximity to the particle. A massive suction can lead to unspecificity and deformation of the particles. P_Release_ is the maximum pressure that is required to induce the release of the particle from pipette. As we lower the pressure values, the particle will be released in a further distance from pipette. A massive injection of pressure will lead to the loss of all liquid from pipette and insertion of air bubbles to sample. (**C**) Catch and release time average and SD. (**D**) Viability of CCL-2 cells after manipulation, i.e., catch and release. Fluorescence intensity of control and manipulated cells colored with PI was measured. The difference between the control population and the manipulated population is not significant $\;P_{value}=0.3151$ $(N_{Manipulated}=156,\;N_{Control}=192$), resulted from Unpaired *t* test with Welch’s correction. (**E**) Positioning error distribution. (**F**) The Euclidian distance between the planed positions to the actual positions of the particles was measured.

The accuracy of the catch and release operations is influenced by the pressure induced. Accurate releasing is more challenging when dealing with the micro-sized particles. For each particle used in the system we defined its *minimum catch pressure*, the minimum pressure that allows the particle to be caught on the pipette. A massive suction, i.e., high pressure values, can lead to the suction of the particle into the pipette, resulting in deformation of the particle and trouble in precise positioning, in the following release (Figure 2B, yellow). In this content, the position of the pipette in close proximity to the desired object before its suction is critical. First, it promises specificity, allows a catch of a single specific particle, rather than a suction of few particles. Secondly, increasing the distance between the pipette and the particle increases the amount of pressure required for the catch of the particle and, in return, will influence the amount of pressure required for its release. When a suction of a distant particle is performed it leads to a suction of the solution and changes the pressure balance. In order to keep the catch and release values identical to all particles, it is important to position the pipette in close proximity to the particle, and avoid a massive suction of solution to the pipette.

Next, the *maximum release pressure* in which the particle is released from the pipette is defined (Figure 2B, blue). As the pressure is decreased, the force on the particle is increased, and the particle is thrown a larger distance from the pipettes tip. The maximum distance a particle can be thrown grows as a function of the amount of medium soaked in the pipette. Therefore, it is better to keep the amount of medium in the pipette in the initial amount (Figure 1G). A massive injection can lead to the loss of all liquid from the pipette, followed by the insertion of air-bubbles into the manipulated sample.

Pressure values are measured by the movement of the microinjector’s fine knob in µm units.

A variety of particles were tested with the system: polystyrene beads, Sepharose beads, agarose hydrogel droplets (synthesized with a droplet-based microfluidic system), and a few types of living cells. Each particle has an individual minimum catch pressure and maximum release pressure that is influenced by its size, material, and density (Table 1).

The assembly process is composed of motions of the pipette and operations, i.e., catch/release (Figure 2a). For each particle in the pattern, the pipette is moved to the particles type area/well and positioned near a particle. The catch operation is performed with pressure set to the minimum catch pressure of the particle. If the particle was not caught, the pressure is increased by 10 µm knob rotation, until the particle is caught successfully. Then, the pipette is moved to the particle’s goal position and the release operation is performed. Pressure is set to the maximum release pressure of the particle. If the particle was not released, the pressure is decreased by a negative 10 µm knob rotation, until the particle is released successfully. At first, the goal task was to rearrange micro objects scattered on the substrate randomly to a given arrangement. The focus was only on arranging objects relative to each other, indicating the accuracy of the patterning, with no care for the absolute location of the arrangement on the substrate.

A total of 56 polystyrene beads were patterned into a square shape. Accuracy and time parameters were measured.

The average time for a bead catch operation is 5.6 s. The time required for an accurate bead release is higher, with an average of 9.7 s (Figure 2C). The time required for an operation can be reduced by adjustment of the speed injection. In addition to the time that is required to catch and release the particles, the global process time includes also the time required for the pipette to move towards catch and release positions. The motion time can be reduced by adjustment of the micromanipulator speed. The global process time, including motion and operations time was measured with an average deposition rate of 1.5 beads/min, i.e., it takes 40 s on average to catch, move, and place a particle. This resultant time can be decreased by full automation of the process. Cells after manipulation, i.e., catch and release, show viability values similar to control cells (Figure 2D), indicating this technique is suitable for living cells.

**Figure 3:**
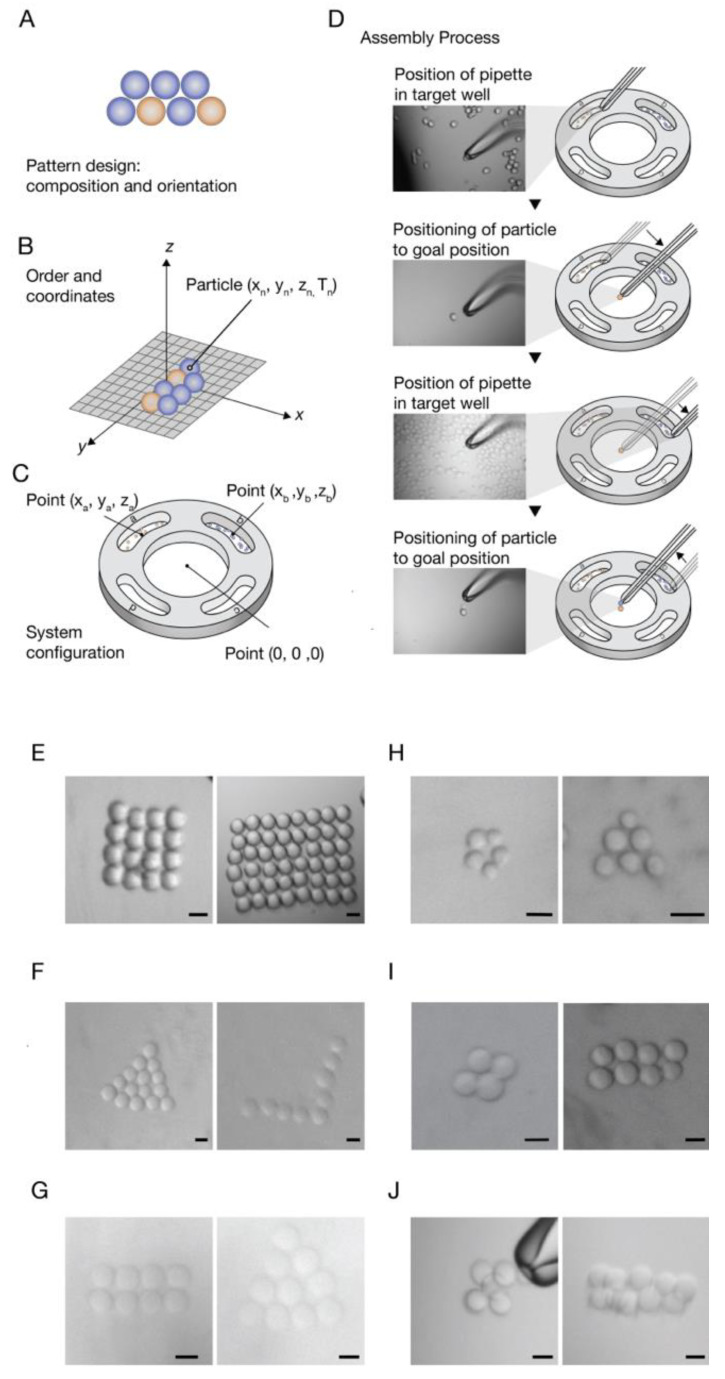
Programmed workflow for patterning of multiple particle types. (**A**) Plan of the pattern design, composition and orientation of the particles. (**B**) Conversion of the pattern design to a coordinate format, i.e., defining the displacement order of the particles positioned in the pattern. Next, the particles coordinators are set in relation to the first particle. (**C**) Defining the coordinate system of the assembly device. The center of the assembly area is set to (0,0,0), and the center of the wells are defined in relation to that point. The first particle in the pattern is placed at that point. (**D**) The assembly process: the micropipette is inserted into an external well, lifts a single particle each time, and releases it at a precise locus in the central well. Showing two types of beads taken from separate side wells and being placed in nearest proximity in the central well. (**E**–**J**) Examples of patterns assembled with our system, for different types of particles: (**E**) polystyrene beads, (**F**) Sepharose beads, (**G**) 3% Agarose droplets. (H) HEP-G2 cells (right) CCL-2 cells (left). (**I**–**J**) Sepharose beads coated with complementary DNA sequences. Patterns form one layer (**I**) and patterns form two layers (**J**). Scale bars: 30 µm.

We present positioning with high precision and accuracy, with an average *positioning* error of 5.3 µm. The positioning error distribution shows that close to 70% of the positioning gave an error smaller than 5 µm, and close to 85% gave an error positioning smaller than 10 µm. (Figure 2E, Video S1). The Euclidian distance between the planned positions to the actual positions of the particles was measured (Figure 2F). Additional 2D patterns were constructed with high accuracy from different particles and cells (Figure 3E–J).

Later, the technique was extended to a programmed pattern technique allowing the displacement of multiple types of particles to form a desired pattern in a targeted defined location.

For this purpose, a CAD prototype of a cell-holding device was designed and printed. The device has separate wells for different particle types and an assembly area. The center round well is where the pattern is built. The four external wells (signed with letters) are for the reservoirs of the different particle types. The cell-holding device is embedded in the working plate (Figure 1D). In this stage, the *minimum air-liquid transition pressure* was defined, i.e., the minimum pressure that is required for the transition of the particles out of the wells (liquid–air transition), and back in to the assembly area (air–liquid transition), without detaching from the pipette (Table 1).

The workflow of a desired pattern composed from multiple particles types starts from the pattern design, which includes particles composition and orientation (Figure 3A). Then, a conversion of the pattern design to a coordinate format is performed, i.e., defining the order of the particles in the patterning process and giving each particle coordinates relevant to the first particle that is set to (X_0_,Y_0_,Z_0_) (Figure 3B). The next step is configuration of the system, which includes setting reservoirs and assembly area center coordinates. The center of the assembly area is determined as the reference (0, 0, 0) point. The first particle in the designed shape will be placed at this point (Figure 3C), giving the particles an absolute position on the substrate. Finally, the assembly process can be started (Figure 3D), as described in the workflow in Figure 2A.

For demonstration of a programmed pattern fabrication composed of multiple particle types, polyesterene beads were loaded in well “A” and sepharose beads were loaded in well “C”. In the assembly area, a basic pattern was constructed from these two types of beads. (Figure 3D, Video S2).

In order to create 3D patterns with our technique, by positioning particles layer by layer, the adhesiveness of the building blocks is required to hold the pattern together. Therefore, we introduced “glue” into our system based on complementary DNA strands [46,47]. Streptavidin Sepharose beads were coated with biotin-modified oligonucleotides through streptavidin-biotin interaction. One population of sepharose beads was coated with sequence *A* and loaded in well “A”, and another population was coated with the complementary sequence *A’*, and loaded in well “C”. The assembly process steps, as described in Figure 3, were performed, for the creation of fixed patterns. When placing an *A* bead near an *A’* bead, they stick to each other. A high positive pressure on the beads can detach them from each other. Patterns created from these DNA coated beads can be seen in Figure 3I,J. Patterns composed form two layers were fabricated with this method, demonstrating the ability to pattern 3D structures as well.

## 4. Discussion

In this study we demonstrated a technique for the active pattering of individual cell-sized particles: beads, droplets, and living cells, with resolution smaller than 10µm, an ability that is missing in the classic positioning strategies, and is important for different single cell experiments and tissue engineering applications. We believe this bottom-up technology can be useful as a platform to investigate cell–cell interactions and cell interactions with external stimuli, such as drugs and growth factors, for the understanding of the processes governing the maturation, cell self-sorting, and the self-assembly capabilities of the cell. Different biological components, such as DNA, protein, or cells, can be encapsulated in agarose (or other hydrogels) droplets [48,49,50,51]. The technique can therefore be used for a variety of experiments. For example, positioning neuronal cells and growth factors encapsulated in droplets in specific locations to guide cell growth. The controlled positioning of neurons with this method can also be helpful in creating a defined neuronal network in a bottom up approach. Another distinct advantage of this technique, is its ability to create colloidal patterns and therefore suitable to build cell-dense tissues with the spatial features the size of single cells, as epithelial tissues.

All the components of the system are motorized and enable a full automation of the whole assembly process. In the patterns presented in this paper; all setting steps were done automatically. Detection steps were done manually (Figure 2A). In order to automate the whole assembly process, image processing should be integrated to handle the detection steps in the process. Full automation will improve performance, accuracy, speed, and enable larger-scale tasks. We offer a file format for patterning in this technique, as it is not limited to specific patterns or shapes, but rather has the potential to work as a 3D printer at single cell resolution. The process is monitored under the microscope, and enables to selectively choose particles, e.g., choosing viable cells upon dead cells or choosing labeled cells by florescence to insert into the pattern.

Adhesion between particles was demonstrated by complementary DNA sequences. This method can be extended to more sequences and types of particles and allow fabrication of complex patterns [45] and 3D patterns. Attachment of these DNA sequences to cells, with no harm to cells [46], was reported. An interesting direction to be investigated in future is controlling interactions between particles by modifying their surfaces with photo switchable proteins, for example, the pair CRY2/CIBN [52]. The induction of light can be done in a single cell resolution by attaching a micro optical fiber to a micromanipulator and controlling its position and operation automatically. Photo switchable “glue” allows adhesion between building blocks, only after the particles locations are assessed as correct, enabling error corrections and a flexible pattern process. In addition to adhesion between particles, the adhesion of particles to the surface can be integrated to the system in the future if needed.

The manipulation of particles and cells 30 µm sized with the SHM was very accurate and easy to use. The BHM handling was more sensitive, as it handles the manipulation of smaller particles, 10–20 µm sized.

The minimum transition air-liquid pressure was not defined for living cells, to avoid unnecessary stress on cells. For patterns composed of multiple cells, an alternative device should be planned that enables separation between cell groups and bypasses the liquid-air transition.

In our study, we presented mainly the assembly of 2D patterns. Merging the cell patterning technique with 3D printing of hydrogel (scaffold), after each layer of cell patterning, could extend the method to 3D fabrication for tissue engineering applications. A means to control the spatial heterogeneity of the extracellular matrix (ECM) is provided by 3D printing, in addition to the spatial heterogeneity of cells provided by our technique. Microinjection and micromanipulation have great potential in the formation of in vitro tissue models for the reproduction and study of micro-architectural characteristics. Practical considerations limit their current application to macro-scale construct production, but these could potentially be combined with macroscale techniques in future applications.

## Figures and Tables

**Table 1:** Calibrated pressure values for all particles. Pressure values were measured by the rotation of the microinjectors fine knob in µm units. Small holding micropipette (SHM) and baby holding micropipette (BHM) were used. Scale bars: 30 µm.

Particle Type	Polystyrene Beads	Sepharose Beads	3% Agarose Droplets	Primary Leech Neurons	CCL-2 Cells	HEP-G2 Cells
**Pipette type**	SHM	SHM	SHM	SHM	BHM	BHM
**Catch Pressure**	50	200	200	200	100	100
**Release Pressure**	−20	−150	−200	−250	−100	−100
**Transition Pressure**	300	600	600	-	-	-
**Diameter**	30 µm	34 µm	20–30 µm	30 µm	20 µm	18 µm
**Particle pattern**	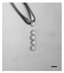	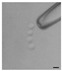	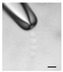	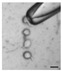	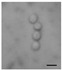	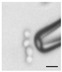

## Supplementary Materials

The following are available online at https://www.mdpi.com/2072-666X/11/5/505/s1, Video S1: Square patterning from one bead type, Video S2: Programmed pattern from two bead types.
